# Risk Factors Contributing to Symptomatic Miniplate Removal following Orthognathic Surgery: Systematic Review and Meta-Analysis

**DOI:** 10.3390/jcm13113335

**Published:** 2024-06-05

**Authors:** Mohamed Jaber, Nadin Abouseif, Mawada Hassan, Alaa Mohamed El-Ameen

**Affiliations:** 1 Department of Clinical Sciences, College of Dentistry, Ajman University, Ajman P.O. Box 346, United Arab Emirates; 201810918@ajmanuni.ac.ae (N.A.); m.abdelmagied@ajman.ac.ae (M.H.); 2 Center of Medical and Bio Allied Health Sciences Research, Ajman University, Ajman P.O. Box 346, United Arab Emirates; 3 College of Science, UAE University, Al Ain P.O. Box 15551, United Arab Emirates; alaajabberr@gmail.com

**Keywords:** complications, orthognathic surgery, osteosynthesis, osteotomy, miniplate, titanium, meta-analysis

## Abstract

**Background/Objectives**: The use of miniplates for stabilizing bones post orthognathic surgery has surged in popularity due to their efficacy in ensuring stability and hastening recovery. However, controversy exists regarding what should be done with these miniplates after surgery. Some surgeons advocate for their removal, while others suggest leaving them in place. This study sought to assess the frequency, causes, and potential risk factors linked with miniplate removal in orthognathic procedures. **Methods**: A thorough meta-analysis was conducted by scrutinizing studies from various databases including PubMed, Google Scholar, Embase, and Scopus, focusing on publications spanning from 1989 to 2023. **Results**: Ten studies meeting the inclusion criteria, encompassing 1603 patients, were chosen for inclusion in the meta-analysis. The male-to-female ratio varied from 0.7:1 to 4:1. Overall, 5595 miniplates were inserted, with 294 (5.3%) being subsequently removed. Primary reasons for miniplate removal included infection (161 cases, 2.9%), exposure of miniplates (34 cases, 0.6%), and palpable plates (23 cases, 0.4%). Other indications comprised pain, patient preference, and temperature sensitivity. Less frequent causes for miniplate removal included sinusitis, secondary surgery, and dental pathology. The mean duration of miniplate removal was 5.5 months, with the majority (56.1%) being removed from the mandible rather than the maxilla. In conclusion, this meta-analysis underscores the importance of miniplate removal when hardware causes complications and physical discomfort. The primary reasons for removing miniplates were infection and plate exposure, with the mandible being the most common removal site. **Conclusions**: These findings emphasize the need for continued monitoring to assess the fate of miniplates in orthognathic surgery and provide valuable information for future clinical decision-making.

## 1. Introduction

Orthognathic surgery, also known as corrective jaw surgery, plays a critical role in the correction of dentofacial deformities and related functional problems. This surgical procedure aims to improve both the aesthetic appearance and functionality of the patient’s face and jaw.

Orthognathic surgery procedures on the upper jaw often involve Le Fort osteotomies, which carry the risk of injury to the maxillary sinus, nasal cavity, or adjacent teeth. Additionally, procedures involving advancement or impaction of the maxilla may impact nasal airflow and speech articulation. In contrast, surgery on the lower jaw commonly entails sagittal split ramus osteotomies or genioplasty. Complications specific to these procedures include injury to the inferior alveolar nerve, mandibular fracture, or relapse of the mandibular position. Furthermore, genioplasty procedures may result in aesthetic asymmetry or chin ptosis if not performed with precision [1,2].

Following these procedures, bone segments are immobilized using fixation plates and screws to establish osteosynthesis, with titanium miniplates and screws being the preferred choice [1,2].

Titanium miniplates and screws have become the standard method for rigid fixation in these surgeries. Traditionally, these miniplates are removed after bone healing, often within 3 months after surgery [3,4,5,6]. The opinions of surgeons on removal varied, with some recommending routine removal and others suggesting it only when necessary. However, the use of titanium, known for its biocompatibility and inertness, has weakened the argument for its routine removal as a foreign body [3,7,8,9].

The literature indicates that a notable percentage of patients undergoing orthognathic surgery may require the removal of plates or screws from the maxilla or mandible. Specifically, removal rates range from 10.6% to 19.2% in the maxilla and from 10% to 24.8% in the mandible. Overall, removal rates for plates placed in either jaw during orthognathic surgery range from 3.6% to 27.5% [3,8,10,11,12]. The wide variation is related to poor study design, heterogeneous samples pooling orthognathic and trauma cases, small sample sizes, non-consecutive cases, and multiple operators. Few studies have eliminated sampling bias by limiting the cohort to only consecutive orthognathic surgery patients.

Commonly reported reasons for the removal of miniplates include infection, exposure of the miniplate, and patient-reported irritation or discomfort [3,8,10,11,12]. The decision to remove miniplates after orthognathic surgery lacks consensus due to associated risks such as infection, morbidity, and cost [3,7,10,12]. This controversy reflects the complexity of balancing potential risks and benefits. While some advocate for routine removal to mitigate complications and optimize long-term outcomes, others argue that selective retention may be safe and effective in certain cases. As the field continues to evolve, further research and consensus guidelines are needed to guide clinical practice and ensure optimal patient care. Thus, the objective of this study was to evaluate the incidence, causes, and possible risk factors associated with the removal of miniplates in orthognathic surgeries and the common complications associated with the removal of miniplates.

## 2. Materials and Methods

This study constitutes a systematic review and meta-analysis. We formulated the research protocol following the guidelines provided by the Preferred Reporting Items for Systematic Reviews and Meta-Analysis Protocols (PRISMA-P) and registered it under the PROSPERO registration number CRD42023399232.

### 2.1. PICO

The search methodology followed the PICO framework, representing P (Patient Population), I (Intervention or Exposure for observational studies), C (Comparison), and O (Outcomes). In this systematic review, we applied the PICO approach, covering the study population (in patients undergoing orthognathic surgery), interventions (titanium miniplate removal), comparisons among (miniplates removed vs. miniplates not removed), and outcomes of interest (the prevalence rate, risk factors, and causes of miniplate removal).

### 2.2. Eligibility Criteria

Inclusion criteria:

1. Full text available
2. Population: Patients who underwent orthognathic surgery.
3. Intervention: Fixation using miniplates.
4. Outcome: prevalence rate, risk factors, and causes of removal of miniplates.
5. Study design: Clinical trials, controlled trials, retrospective and prospective studies, and case series.

Exclusion Criteria:

1. Incomplete data: Studies that lack essential data on complications in orthognathic surgery were excluded to ensure the reliability and comprehensiveness of the findings.
2. Non-English language: studies published in languages other than English were excluded.
3. Non-peer-reviewed literature: including conference abstracts, posters, and unpublished studies, was excluded to maintain the quality and rigor of the evidence included in the review.
4. Animal studies: Studies conducted in animal models were excluded because they may not directly translate into human outcomes and may not adequately reflect the complications experienced by human patients undergoing orthognathic surgery.
5. Non-relevant population: Studies focusing on populations not representative of orthognathic surgery patients, such as those with congenital craniofacial anomalies or trauma-related injuries, were excluded to ensure the relevance of the findings to the target population.
6. Pediatric patients: Studies exclusively involving pediatric patients were excluded due to differences in anatomical considerations, surgical techniques, and complication profiles compared to adult populations undergoing orthognathic surgery.
7. Specific medical comorbidities: Studies focusing solely on patients with specific medical comorbidities, such as severe cardiovascular disease or uncontrolled diabetes, were excluded to maintain the homogeneity of the study population and reduce confounding factors affecting the rates of complication.
8. Publication date: Studies published before a specified date were excluded to ensure the inclusion of the latest evidence available at the time of review.
9. Duplicate studies: Duplicate publications or redundant data from the same study cohort were excluded to avoid duplication of results and ensure the integrity of the analysis.
10. Articles without complete demographic information.
11. Follow-up was less than 6 months.

### 2.3. Information Sources

Following the guidelines outlined in the PRISMA statement, we conducted an electronic search of various databases, including PubMed, Google Scholar, Embase, and Scopus. We also performed a search for gray literature using Open Gray version 1 and manually checked the references in the identified articles. We included studies published between 1989 and 2023 in our meta-analysis.

### 2.4. Search Strategy and Article Selection

To identify studies related to orthognathic surgery, we used these terms: corrective jaw surgery, orthognathic surgery, bilateral sagittal split osteotomy, Le Fort I osteotomy, genioplasty, and jaw surgery. For plate removal, we used the following terms: plate removal, plates removal, plate failure, plates failure, titanium miniplates, screw failure, complication, and removal. We used the Boolean operators ‘OR’ to broaden the search and ‘AND’ to combine different areas. The search equations for each database were as follows:

Search strategy to identify studies in primary electronic databases:

- PubMed: (“corrective jaw surgery”) OR (“orthognathic surgery”) (“Bilateral Sagittal Split osteotomy”) OR (Le Fort I osteotomy) OR (“Genioplasty”) AND (“plate removal” OR “plates removal” OR “plate failure” OR “plates failure”).
- Scopus: (corrective jaw surgery) OR (orthognathic surgery) (“bilateral sagittal split osteotomy”) OR (“Le Fort I osteotomy”) OR (“Genioplasty”) AND (“plate removal” OR “plates removal” OR “plate failure” OR “plates failure”).
- Embase: (“corrective jaw surgery”) OR (“orthognathic surgery”) (“Bilateral sagittal split osteotomy”) OR (“Le Fort I osteotomy”) OR (“Genioplasty”) AND (“plate removal” OR “plates removal” OR “plate failure” OR “plates failure”).
- Google Scholar: (“corrective jaw surgery”) OR (“orthognathic surgery”) (“Bilateral sagittal split osteotomy”) OR (“Le Fort I osteotomy”) OR (“Genioplasty”) AND (“plate removal” OR “plates removal” OR “plate failure” OR “plates failure”).

The studies selected for analysis were those that reported the results of miniplate use in orthognathic surgery, including details on complications and removal rates.

Two independent reviewers, NA and MH, evaluated the titles and abstracts of all studies found in the initial search. If the abstracts did not provide sufficient information, the reviewers examined the full text to determine whether to include or exclude the studies. The authors reviewed the full texts of all remaining articles. Any discrepancies in the results between the reviewers were resolved by consensus, and if agreement could not be reached, a third researcher (MJ) was consulted.

### 2.5. Data Collection Process

We systematically examined all pertinent research articles concerning the extraction of miniplates in orthognathic surgery patients. Two independent evaluators gathered the following information from eligible articles: author(s) name, year of publication, study design, number of patients and plates implanted, mean patient age, gender distribution, miniplate placement site (mandible or maxilla), rationale for miniplate removal, and average follow-up duration in months. To gauge the agreement level between these evaluators, the kappa statistic was employed, utilizing the same criteria utilized during the study selection phase. Any disparities were resolved through discussion between the evaluators; if consensus couldn’t be reached, a third assessor (MJ) was consulted to provide input.

### 2.6. Evaluation of the Study Risk of Bias

We utilized the Newcastle–Ottawa Scale (NOS) to evaluate the quality of the chosen studies, assigning them into categories of “good”, “fair”, or “poor”. Studies of high quality obtained 3 or 4 stars in the selection domain, 1 or 2 stars in the comparability domain, and 2 or 3 stars in the outcome/exposure domain. Those categorized as fair quality garnered 2 stars in the selection domain, 1 or 2 stars in the comparability domain, and 2 or 3 stars in the outcome/exposure domain. Studies classified as poor quality received 0 or 1 star in the selection domain, 0 stars in the comparability domain, or 0 or 1 star in the outcome/exposure domain. The resultant classification (good, fair, or poor) reflected the overall study quality based on these criteria. One study [13] was deemed ‘fair’, while all other studies were considered of good quality, as depicted in Table 1.

### 2.7. Statistical Analysis

Data were analyzed with comprehensive meta-analysis software (CMA-V4). The random-effects model was used to estimate the pooled prevalence and 95% confidence intervals (CI). The heterogeneity between studies was evaluated using Cochran’s Q statistic and the I^2^ statistic, which describes the percentage of total variation between included studies due to heterogeneity, as well as the tau-squared (T^2^) test.

To further interpret the results of the meta-analysis, we also performed a sensitivity analysis by removing one study at a time and evaluating the impact on the overall results. This analysis helps to assess the robustness of the findings and the potential influence of any individual study on the overall estimate of the effect. The sensitivity analysis showed that the estimate of the overall effect was not significantly affected by the removal of any individual study. The results remained consistent and the effect size was within the range of the overall estimate.

In addition, we assessed the heterogeneity of the included studies using a chi-square test and the I-square statistic. The chi-square test result was significant (*p* < 0.001), indicating significant heterogeneity between the studies. To explore possible sources of heterogeneity, we performed a subgroup analysis based on the type of indication for plate removal.

## 3. Results

**Figure 1:**
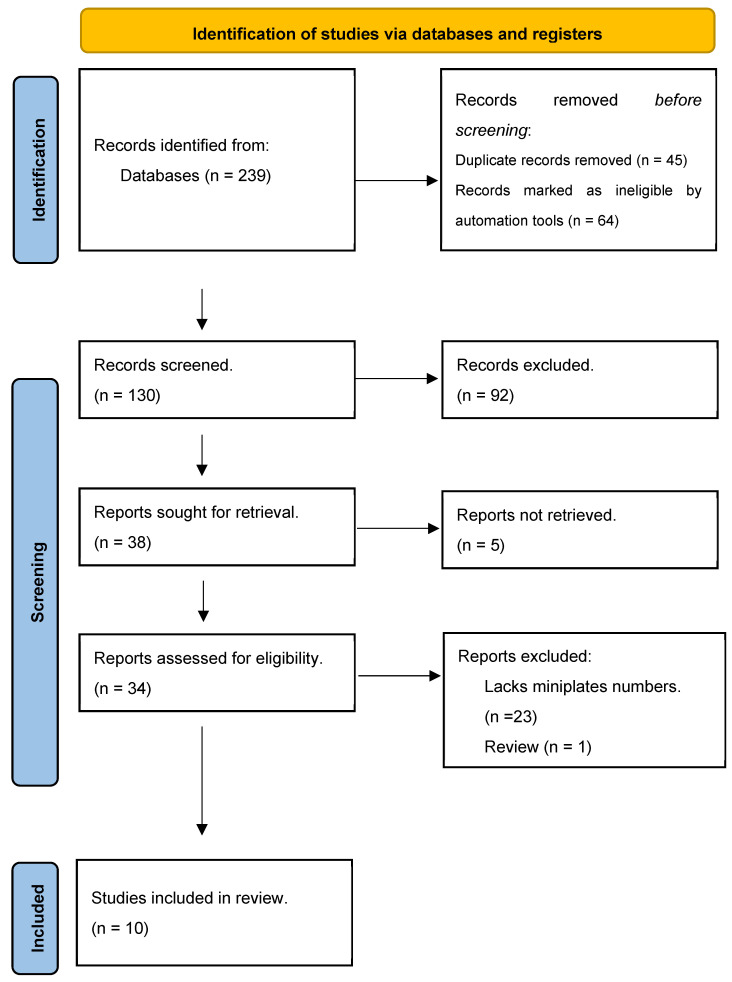
Preferred Reporting Items for Systematic reviews and Meta-Analyses (PRISMA) flow diagram.

The review included 10 articles that met our inclusion criteria and were published between 1989 and 2023. The initial database search retrieved 239 articles. There were 45 duplicates and 64 articles were excluded. The titles and abstracts of the remaining 130 articles were scanned, leading to the exclusion of 92 articles. The 38 remaining potentially eligible articles were identified and only 34 were recovered. After a second round of full-text screening, 10 articles met the eligibility criteria and were included in this systematic review and meta-analysis (Figure 1).

A risk of bias assessment was performed following the Newcastle–Ottawa Scale (Table 1). Ten studies with a total of 1603 patients were evaluated. The M:F ratio ranges from 4:1 (O’Connell et al. [6]) to 109:141 (Ulker et al. [18]). Most of the studies were retrospective in nature, with the largest study including 533 patients (Alpha et al. [2]) (Table S1). A total of 5595 miniplates were inserted and the total number of miniplates removed was 294 (5.3%). The most common reason for the removal of miniplates was infection, in 161 patients (2.9%), followed by exposure to miniplates (34 patients, 0.6%) and palpable plates (23 patients, 0.4%). Other indications included pain (19 patients, 0.3%), patient preference (16 patients, 0.3%), and temperature sensitivity (14 patients, 0.2%). Other relatively less common reasons for the removal of miniplates included sinusitis, a second surgery, and dental pathology (Table S2).

The duration of miniplate removal varies between studies, with some authors reporting a mean duration of 5.5 months, while others reporting a duration of up to 72 months. For example, Theodossy et al. [11] reported a mean duration of 5.5 months, while Cubuk et al. [17] reported a mean duration of 6 years.

The removal site of the miniplates also varied; for example, some studies have reported a higher incidence of removal in the mandible (147, 6.5%) than in the maxilla (115, 4.5%) (Table S3).

### 3.1. Meta-Analysis of the Selected Articles

**Figure 2:**
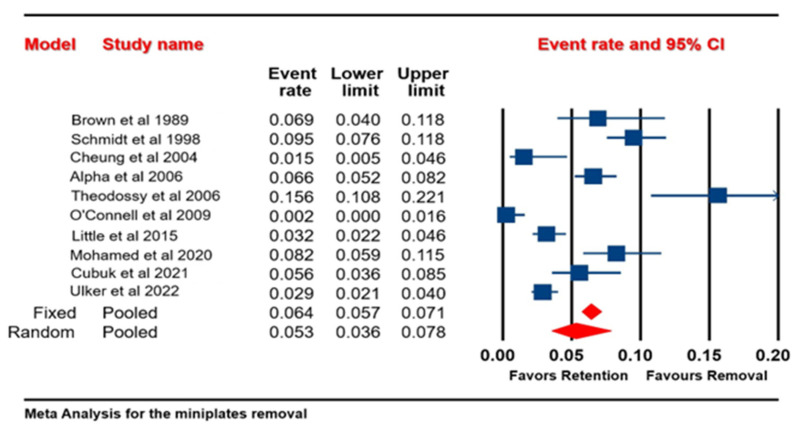
Forest plot of all selected studies showing the percentage of patients who underwent miniplate removal in both the maxilla and the mandible [2,6,10,11,13,14,15,16,17,18].

The analysis is based on ten studies. The effect size index is the event rate. A random-effects model was used for the analysis. The studies in the analysis are assumed to be a random sample of a universe of potential studies, and this analysis was used to make an inference to that universe. The mean effect size was 0.053, with a 95% confidence interval of 0.036 to 0.078. The mean effect size in the cohort of comparable studies could fall anywhere in this interval. The I-squared statistic is 90%, indicating that 90% of the variance in observed effects reflects the variance in true effects rather than the sampling error. The meta-analysis revealed that the odds of removing the miniplates were 0.053 times greater than the odds of retaining the miniplates (Figure 2). The terms “favors retention” and “favors removal” imply that lower event rates may be preferable for retention, while higher rates favor removal.

**Figure 3:**
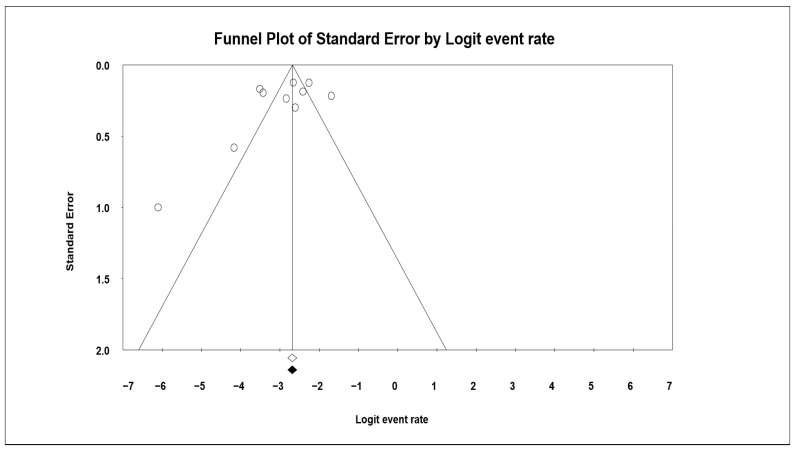
Funnel plot of all selected studies showing the percentage of patients who underwent miniplate removal in both the maxilla and the mandible.

A funnel plot was generated that measures the study size (usually standard error or precision) on the vertical axis as a function of the effect size on the horizontal axis. Large studies appear toward the top of the graph and tend to cluster near the mean effect size. Smaller studies appear toward the bottom of the graph, and since there is more sampling variation in effect size estimates in smaller studies, they will be dispersed across a range of values (Figure 3).

### 3.2. Miniplate Removal in the Mandible and the Maxilla

This analysis is based on five studies. The effect size index was the odds ratio (OR). A random-effects model was used for the analysis. The studies in the analysis are assumed to be a random sample of a universe of potential studies, and this analysis was used to make an inference to that universe.

**Figure 4:**
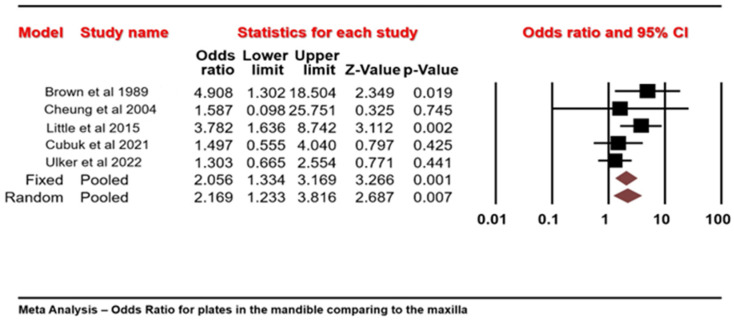
Forest plot of five selected studies showing the percentage of patients who underwent miniplate removal when the maxillary to mandibular arches were compared (odds > 1 favor the removal of miniplates in the mandibular arch more than in the maxillary arch) [10,14,15,17,18].

The Q-statistic provides a test of the null hypothesis that all studies in the analysis share a common effect size. The Q value is 5.873 with 4 degrees of freedom and *p* = 0.209. Using a criterion alpha of 0.100, we cannot reject the null hypothesis that the true effect size is the same in all these studies. The meta-analysis revealed that the odds of removing miniplates in the mandible were 2.169 times greater than the odds of removing miniplates in the maxilla, with an acceptable heterogeneity of 32%, based on the I-squared statistics (Figure 4).

The forest plot also suggested that on average the event rate for miniplate removal was low, with both fixed- and random-effects models showing less than 10% of such events, which statistically favored retention of the miniplates.

## 4. Discussion

The use of miniplates to stabilize bones has gained popularity due to their efficacy in achieving stability and promoting rapid recovery, but these methods present several challenges, which include growth inhibition, palpable presence of plates and screws, difficulties with imaging and radiation therapy, temperature sensitivity, dispersion of titanium particles in lymph nodes, bone stress due to rigidity of the system, and the potential risk of genetic mutations. Consequently, approximately one-third of these systems are removed, leading to increased costs and additional burdens [1,2,3,4,5,6,7,8].

In this study, we evaluated a total of 1603 patients spanning different age groups, with a predominance of female participants. A total of 5595 miniplates were inserted into the mandible and maxilla, and we observed a 5.3% incidence of miniplate removal after orthognathic surgery. The ten selected studies were conducted in various countries and could reflect divergent healthcare practices and demographics of patients.

Inconsistency in the literature on the influence of patient age on the need for miniplate removal suggests a complex interplay of factors that can affect surgical outcomes [11,19]. In this study, we found higher removal rates among patients under 40 years of age than among patients under 30 years of age. This is in agreement with other reported studies [11,14,19].

We observed gender differences, with women being more likely to have their miniplates removed. This may be due to behavioral or physiological differences that affect postoperative recovery and the need for additional procedures. Manor et al. suggested that this could be because women are more willing to seek medical attention for symptoms such as pain, sensitivity, and palpability [19].

This study did not investigate how medical conditions influence miniplate removal due to insufficient data in the reviewed studies, highlighting the need for more extensive research. Previous studies strongly cautioned against smoking before orthognathic surgery, as it could increase the risk of infection among smokers, who were significantly more likely to require miniplate removal [11,12,19,20]. These findings emphasize the importance of following preoperative instructions to mitigate postoperative complications.

In the current study, miniplate infection and exposure were the most common causes of miniplate removal, similar observations were made by other researchers who also identified infection as the leading cause of miniplate removal [2,3,11,12]. Miniplates, located in sensitive submucosal tissues, are vulnerable to external trauma and masticatory forces. These factors can destabilize miniplates, leading to screw-loosening, inflammation, and an increased risk of infection. Furthermore, improper suturing techniques and insufficient bone cooling during screw hole preparation have been associated with miniplate failures due to infections [2,3,11,12]. Miniplate-associated infections can arise from bacteria in the oral cavity or improper sterile practices during surgery. Sometimes, these infections are related to dental damage during the fixation procedure or to decreased blood flow in the mandible. However, if the infection does not involve bone, it is possible to retain the miniplates by administering antibiotics, excising infected tissue, and tackling the root cause of the infection. Studies suggest that smaller plates can become reservoirs for bacteria, leading to persistent inflammation and discomfort, indicating an increased risk of infection the longer a plate remains in the body [21,22,23]. Alpha et al. [2] reported that 6.5% of the miniplates (70 out of 1066) were removed due to symptoms such as erythema, fistula, granulation tissue, hematoma, or wound dehiscence, which were classified as ‘disturbances in healing’. Similarly, Mohamed et al. [16] noted plate removal due to infection, with patients showing various signs, such as sinus tracts, localized swelling, sinusitis, and superficial infections. Theodossy et al. [11] observed a 15.6% removal rate, with all removed plates attributed to infections, manifesting as pain, swelling, wound dehiscence, and pus discharge.

As in several other investigations [2,3,6,8,9,10,11,12,14], we recognized additional reasons for miniplate removal, including pain, plate exposure, growth limitations, and requirements for prosthetic rehabilitation. The discrepancies observed in different studies underscore the intricate character of orthognathic surgeries. These disparities explain how individual patient factors shape the decision-making process regarding plate removal. Furthermore, these distinctions highlight the crucial requirement for thorough patient evaluations and tailored approaches in these surgical interventions [1,6,21,22,23].

In this study, pain accounted for 0.3% (19 patients) of miniplate removal. Miniplates can cause discomfort or pain, particularly when placed near facial muscles, nerves, or tendons. This discomfort can significantly affect the quality of life, especially if the miniplate is visible on the cheek or jawbone [24]. Pain can arise from various sources, including nerve damage, plate migration, or cold intolerance. Brown et al. [14], reported that several patients complained of pain in the area surrounding the plates placed, and symptoms were relieved when the plates were removed. Similarly, Little et al. [10] reported that patients who complained of pain had no associated infection or plate exposure to explain pain. Schmidt et al. [13] reported that pain was the most common reason for miniplate removal in their study, occurring alone or with other symptoms.

Miniplates can sometimes be visible under the skin or cause scarring, raising cosmetic concerns for certain patients and causing them to opt for removal. Plate exposure occurs when the miniplate becomes visible through the skin or mucosa due to inadequate wound closure, inadequate tissue coverage, or a suboptimal plate position. Cheung et al. [15] reported that the incidence of plate exposure for titanium miniplates was 1.02%, with 2 plates removed due to exposure, mainly in the posterior maxilla and mandibular premolar region, attributed to thin mucosa and Le Fort I cut. Mohamed et al. [16] indicated that the primary reason for removal was exposure to plates (11 out of 31 plates), with no pain or infection accompanying exposure. Schmidt et al. [13] reported that plates placed in the maxillary buttress region were removed due to palpation. The authors recommended the use of lower profile plates in these regions to reduce palpability and suggested the use of absorbable plates as an alternative. Sinusitis may occur as a rare complication after Le Fort I osteotomy and is related to disturbance of the sinus mucosa due to the placement of the plate, as reported by Schmidt et al. [13]. Likewise, Little et al. [10] and Ulker et al. [18] reported that only one and two plates, respectively, were removed due to sinus infection.

Other reported reasons for the removal of miniplates include dental implant prosthetic rehabilitation, miniplate fractures resulting from improper plate placement or excessive stress, loose fixation devices due to suboptimal plate placement, improper screw positioning, patient requests, poor aesthetics, nerve impairment, and cancer concerns. Some studies have also reported that patients experience temperature sensitivity in the region around plates, particularly intolerance to cold. For example, Cubuk et al. [17] described patients experiencing discomfort during cold weather, leading to plate removal for relief, and Cubuk et al. [17] documented patients who required the removal of maxillary plates while undergoing additional surgical procedures such as rhinoplasty and sinus lift.

Although titanium-based alloy miniplates are generally recognized for their benefits in osteosynthesis, uncertainties persist regarding their long-term effects. Recent research has focused on evaluating tissue around removed titanium materials to assess potential impacts [25,26]. Some authors reported about the local cellular effects of metallic particles, which can infiltrate nearby tissue and potentially may travel through the lymphatic system to other organs. Studies have indicated local reactions and immune-inflammatory responses in fibrous connective tissue deposits, with titanium inducing oxidative stress and allergic reactions; in addition, instances of facial eczema have been reported [25,26].

The timing for miniplate removal exhibits variability, with the majority of cases taking place within one year following the initial surgery. The literature indicates that removal frequently happens between 6 months and 1 year after fixation, with certain instances even occurring in less than 3 months. Generally, miniplates are extracted after ensuring bone healing, typically within 6 months to 1 year. Factors such as patient-specific considerations, surgical methods, rates of healing, and potential complications contribute to the diverse range of removal timelines observed in this study. Tailored patient management and ongoing assessment are crucial for determining the most suitable timing for removal. The use of operating time as a risk factor has been debated, with extremes of less than 100 and more than 190 min cited as increasing risks [9]. Shorter durations could indicate rushed surgeries with poor placement of the miniplate, while longer durations could indicate prolonged exposure to oral microflora [10,19].

In this study, most of the miniplates (56.1%) were removed from the mandible. The relatively dense structure of the cortical bone of the mandible and the risks associated with surgical interventions likely contributed to this pattern. Furthermore, the thickness of the intraoral mucosa during surgical approaches increases the probability of plate exposure. In the case of the mandible, which withstands more biomechanical forces than the maxilla [27,28], problems such as loose screws and inflammation are more prevalent, especially in osteotomies lacking interfragmentary stability [29]. The distinct vascular supply and structure of the mandible can also impact the effectiveness of these fixation systems [29].

Gareb et al. [29] compared the long-term clinical performance of titanium and biodegradable plates in the fixation of maxillary, mandibular, and bimaxillary osteotomies and concluded that there were no notable differences in the frequency of removal of symptomatic plates between these types. However, there was a tendency to prefer biodegradable osteosyntheses for maxillary osteotomies, while titanium systems were more common for mandibular and bimaxillary osteotomies. This finding suggested that biodegradable options might be more appropriate for maxillary osteotomies, possibly due to lower rates of removal of symptomatic plates. Future research could include the impact of factors such as distance from skeletal movement, oral hygiene, and proximity to incision sites on miniplate removal. Similarly, Ueki et al. [30], using a randomized controlled trial, reported that 25% of titanium osteosyntheses patients developed issues with TMJ function one year after surgery, and some experienced long-term impairment of mandibular function; therefore, future studies should include evaluations of TMJ function using validated questionnaires for a complete assessment.

Cost considerations also play an important role in the question of whether to remove miniplates after orthognathic surgery healing, with substantial variations in removal costs between different countries. Van Bakelen et al. [31] conducted a randomized controlled trial (RCT) and evaluated the general financial burden on patients admitted for removal of titanium miniplates; the authors found that the average total costs were significantly high at the 2-year follow-up. These costs were attributed to a variety of factors, including perioperative expenses, costs of additional medical procedures, travel expenses, and economic impact due to absence from work. This detailed cost evaluation offers a broader understanding of the economic impact of the use of titanium osteosynthesis systems in orthognathic surgeries.

Despite the systematic approach used in the review, based on the registered protocol and adherence to the PRISMA statement, some limitations persisted. The comprehensive and updated literature search was limited by the inability to retrieve some data, which were not included in the primary studies. This gap underscores the importance of complete data reporting in clinical studies. The heterogeneity observed in the studies was attributed to differences in the types of osteosynthesis systems used, variations in surgical procedures, and differences in operative displacement. Therefore, future research should follow strict protocols, focusing on well-defined inclusion and exclusion criteria and specific endpoints to minimize reporting bias. Longer follow-up periods are also recommended to better assess the severity of symptoms caused by the miniplates and the stability of the skeletal system. Clear definitions and adherence to indications for device removal are crucial in reducing detection bias.

In conclusion, this meta-analysis underscores the importance of miniplate removal when hardware causes complications and physical discomfort. The primary reasons for removing miniplates were infection and plate exposure, with the mandible being the most common removal site. These findings emphasize the need for continued monitoring to assess the fate of miniplates in orthognathic surgery and provide valuable information for future clinical decision-making.

## Figures and Tables

**Table 1:** Quality of the selected studies according to the Newcastle–Ottawa scale (NOS).

Author/Year	Selection				Comparability	Outcomes			Score
	Representativeness of the Exposed Cohort	Selection of the Non-Exposed Cohort	Ascertainment of Exposure	Demonstration That Outcome of Interest Was Not Present at Start of Study	Comparability of Cohorts on the Basis of the Design or Analysis	Assessment of Outcome	Was Follow-Up Long Enough for Outcomes to Occur	Adequacy of Follow-Up of Cohorts	
Brown et al., 1989 [14]	★	★	★	★	★★	★	★	★	9
Schmidt et al., 1998 [13]	★	☆	☆	★	★☆	★	★	★	6
Cheung et al., 2004 [15]	★	★	★	★	★☆	★	☆	★	7
Alpha et al., 2006 [2]	★	☆	★	★	☆☆	★	★	★	7
Theodossy et al., 2006 [11]	★	★	★	★	★☆	★	★	★	8
O’Connell et al., 2009 [6]	☆	★	★	★	★☆	★	★	★	7
Little et al., 2015 [10]	★	★	★	★	★★	★	★	★	9
Mohamed et al., 2020 [16]	★	★	★	☆	★☆	★	★	★	8
Cubuk et al., 2021 [17]	★	☆	★	★	★☆	★	★	★	7
Ulker et al., 2022 [18]	★	★	★	★	★★	★	★	★	9

The Newcastle–Ottawa Scale uses black stars (★) to signify that a study satisfactorily meets a specific criterion. The cumulative number of black stars denotes the overall quality of the study. Conversely, white stars (☆) indicate that a criterion is not met, which lowers the study’s quality score.

## Data Availability

Not applicable.

## Supplementary Materials

The following supporting information can be downloaded at: https://www.mdpi.com/article/10.3390/jcm13113335/s1, Table S1: Quality of the selected studies according to the Newcastle–Ottawa scale (NOS).; Table S2: Studies characteristics; Table S3: Reasons for removal of the mini plates; Table S4: Miniplates inserted and miniplates removed in mandible and maxilla.
