# Supplementary material for: Risk Factors Contributing to Symptomatic Miniplate Removal following Orthognathic Surgery: Systematic Review and Meta-Analysis
Source: J Clin Med. 2024 Jun 5;13(11):3335. doi: 10.3390/jcm13113335 (PMC11172665; doi:10.3390/jcm13113335)
Supplement: Supplementary file 1 [file jcm-13-03335-s001.zip › jcm-2991725-supplementary.pdf]

Table S1. Studies characteristics

Author/ Year	Country	# of pts	Age range (yrs)	M:F ratio	Site of plate placement	Type of plates	# of plates	Surgical approach	Reasons for plate removal	Duration Between insertion & removal	Site of plate removal
Brown et al, [13]	UK	47	15-80	-	Maxilla<br>- Buttress<br>- Nasal bone<br>- Piriform<br>Mandible<br>- Angle<br>- Symphysis	Stainless steel	174	Intraoral	• Infection<br>• Pain	3 - 24months	Maxilla<br>- Buttress<br>- Nasal bone<br>- Piriform<br>Mandible<br>- Angle
Schmidt et al., [15]	US	190	Avg 25.42 ± 9.49	72:118	-	Titanium<br>Stainless steel	738	-	• Palpation<br>• Sinusitis<br>• Pain/infection<br>• Pain/temp sensitivity<br>• Pain/palpation<br>• Pain<br>• Infection<br>• Palpation/temp sensitivity<br>• request	2-43 months<br>Avg: 13.7 months	Mandible<br>Maxillary buttress region
Cheung et al., [14]	Hong Kong	30	16-37	1:2.3	Mandible<br>Maxilla	Titanium	196	-	• Infection<br>• Plate exposure	0-24 months	Mandible<br>Maxilla
Alpha et al., [2]	California	533	13-68<br>Avg: 27.6	179:354	Mandible	Titanium	1066	-	• infection	-	Mandible
Theodossy et al., [11]	UK	80	15-50<br>Avg: 25	1:2.8	Mandible	Titanium	160	Intraoral	• Infection	2-12 months<br>Avg: 5.5 months	Mandible

O'Connell et al., [6]	Ireland	101	3-72	-	Maxilla	Titanium	447	-	• Infection	2.5-68 months<br>Avg: 19 months	Maxilla
Little et al., [10]	UK	202	16.9-40.7<br>Avg: 24.6	73:129	Maxilla<br>Mandible	Titanium	854	-	• Exposure<br>• Infection<br>• Palpability<br>• Pain<br>• Dental pathology<br>• Sinusitis<br>• Unexplained swelling	84 - 1071 days<br>Avg: 281 days	Mandible<br>Maxilla
Mohamed et al., [16]	India	100	17-31<br>Avg: 21.33	50:50	Maxilla<br>Mandible	Titanium	376	-	• Exposure<br>• Infection<br>• Request<br>• Pain	6-61 months<br>Avg: 22.33 months	Maxilla<br>Mandible
Cubuk et al., [17]	Ankara	70	Avg: 23.75 ± 6.37	30:40	Maxilla<br>Mandible<br>Chin	Titanium	342	-	• Infection<br>• Cold sensitivity<br>• Palpability<br>• Request<br>• 2nd operation	4 months - 6 years<br>Avg: 1.52 years	Maxilla<br>Mandible
Ulker et al., [18]	Istanbul	250	17-55<br>Avg: 22.9 ± 6.5	109:141	Mandible<br>Maxilla	Titanium	1242	-	• Exposure<br>• Infection<br>• Sinusitis<br>• Request<br>• Secondary surgery<br>• Dental pathology	3-34 months<br>Avg: 11.21± 8.21 months	Maxilla<br>Mandible
Total	-	1603	3-80	1:1.8	Maxilla<br>Mandible		5595			0-72 months	Maxilla<br>Mandible

Table S2. Reasons for removal of the mini plates

Author/Year	Number of plates	Indications for plate removal										Total No
		Infection	Sinusitis	Plate exposure	Temperature sensitivity	Pain	Secondary surgery	Palpable plate	Dental pathology	Patients' preference.	Other reasons	
Brown et al, [13]	174	9	-	-	-	3	-	-	-	-		N= 12
Schmidt et al., [15]	738	16	10	-	12	8	-	20	-	4		N=70
Cheung et al., [14]	196	1	-	2	-	-	-	-	-	-		N=3
Alpha et al., [2]	1066	70	-	-	-	-	-	-	-	-		N=70
Theodossy et al., [11]	160	25	-	-	-	-	-	-	-	-		N= 25
O'Connell et al., [6]	447	1	-	-	-	-	-	-	-	-		N=1
Little et al., [10]	854	8	1	11	-	4	-	1	1	-	Unexplained swelling: 1	N=27
Mohamed et al., [16]	376	10	-	11	-	4	-	-	-	6		N=31
Cubuk et al., [17]	342	10	-	-	2	-	3	2	-	2		N=19
Ulker et al., [18]	1242	11	2	10	-	-	7	-	2	4		N=36
Total	5595	161 (2.9%)	13 (0.2%)	34 (0.6%)	14 (0.2%)	19 (0.3)	10 (0.1%)	23 (0.4%)	3(0.05%)	16 (0.3%)	Other: 1(0.01%)	294 (5.3%)

Table S3. Miniplates inserted and miniplates removed in mandible and maxilla*

**Author/ Year**	Total number of mini plates placed (both jaws)	The total number of mini plates removed (both jaws)	No of miniplates placed in (mandible)	No of miniplates removed from (mandible).	No of mini plates placed (maxilla)	No of mini plates removed from (maxilla)
Brown et al, [13]	174	12 (6.9%)	21	4 (19%)	153	8 (5.2%)
Schmidt et al., [15]	738	70	-	-	738	70 (9.5%)
Cheung et al., [14]	196	3 (1.5%)	76	1 (1.3%)	120	2 (1.6%)
Alpha et al., [2]	1066	70	1066	70	-	-
Theodossy et al, [11]	160	25	160	25	-	-
Little et al., [10]	854	27	338	19	516	8
Cubuk et al., [17]	342	19	138	13	204	6
Ulker et al., [18]	1242	36	442	15	800	21
Total	4772	262 (5.5%)	2241	147 (6.5%)	2531	115 (4.5%)

- Two studies (O' Connell et al [6] and Mohamed et al, [16]) were excluded because of a lack of details
